# Supplementary figures and images for: METALIC reveals interorganelle lipid flux in live cells by enzymatic mass tagging
Source: Nat Cell Biol. Author manuscript; Available in PMC 2022 Jun 24. (PMC9203272; doi:10.1038/s41556-022-00917-9)

Extended Data Figure 5

Blot: mCherry

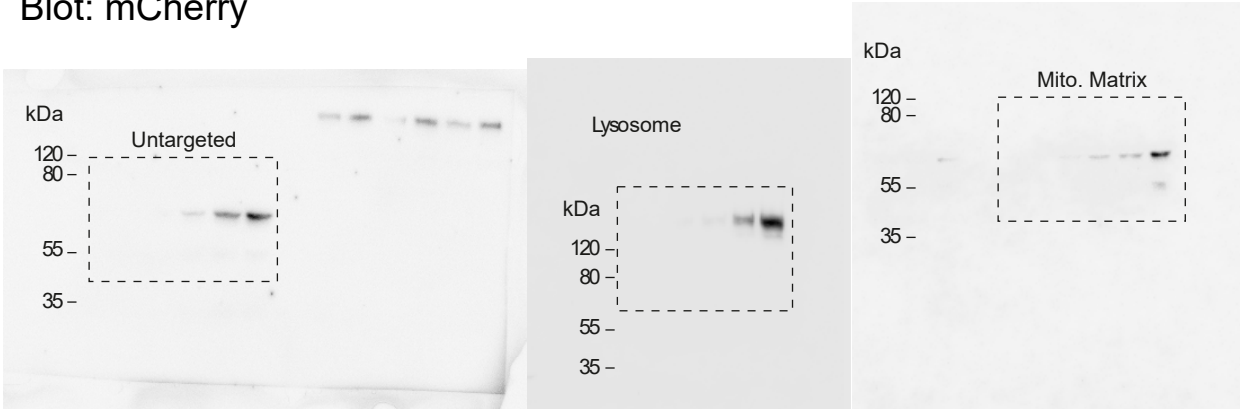

Ponceau

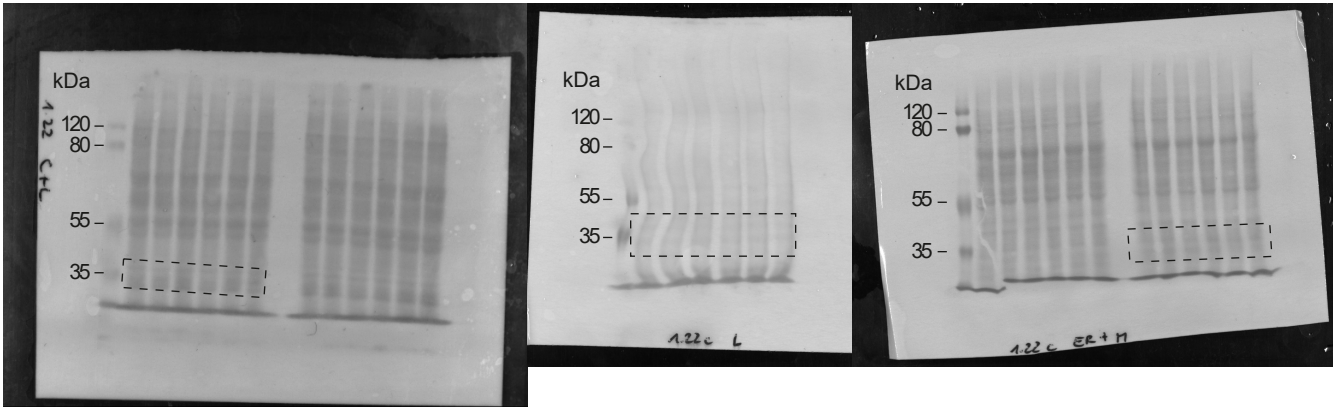

Blot: mCherry

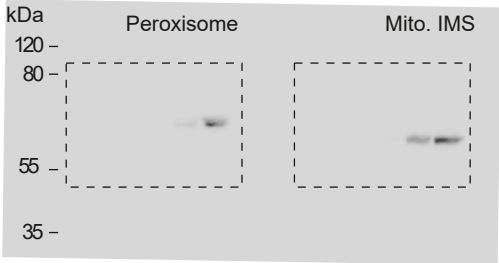

Ponceau

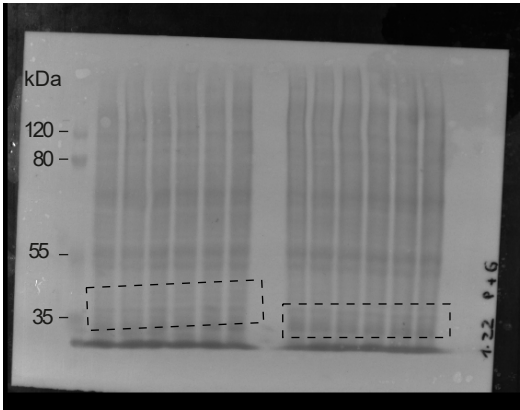

Supplement: Source Data Extended data Figure 5 [file EMS144463-supplement-Source_Data_Extended_data_Figure_5.pdf]

Figure 2a

Blot: mCherry

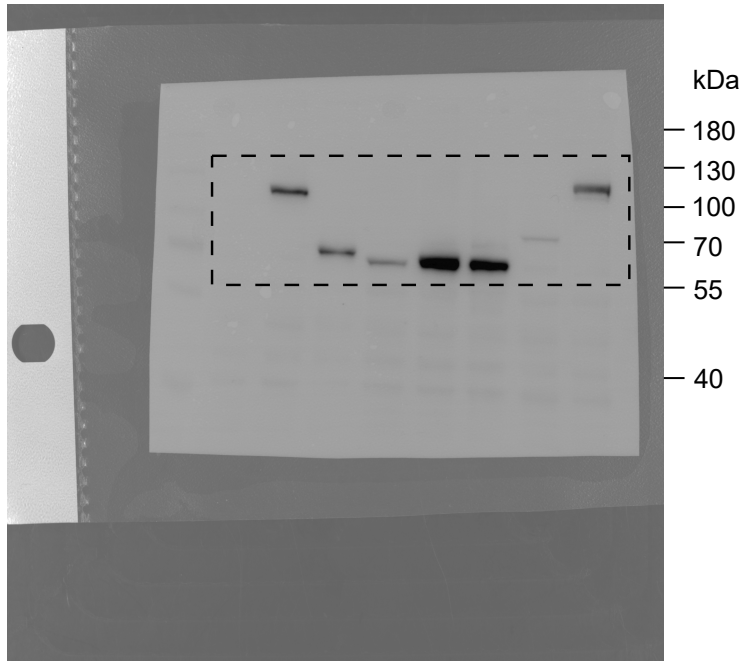

Blot: Pgk1

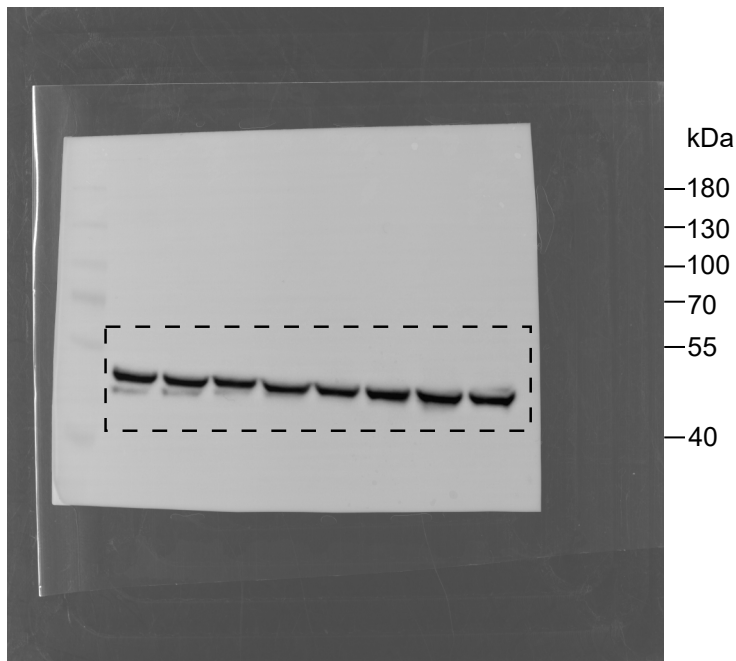

Supplement: Source Data Figure 2 [file EMS144463-supplement-Source_Data_Figure_2.pdf]

Figure 4a

Blot: Flag

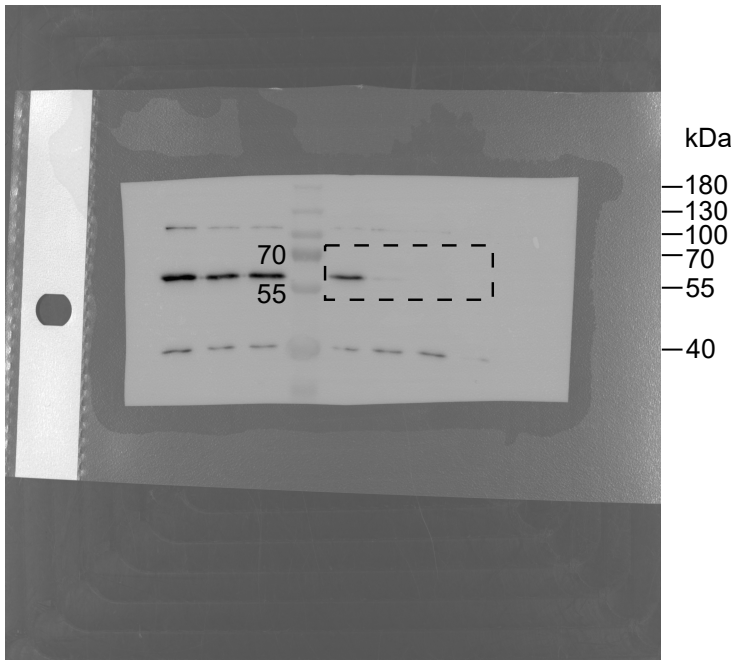

Blot: Pgk1

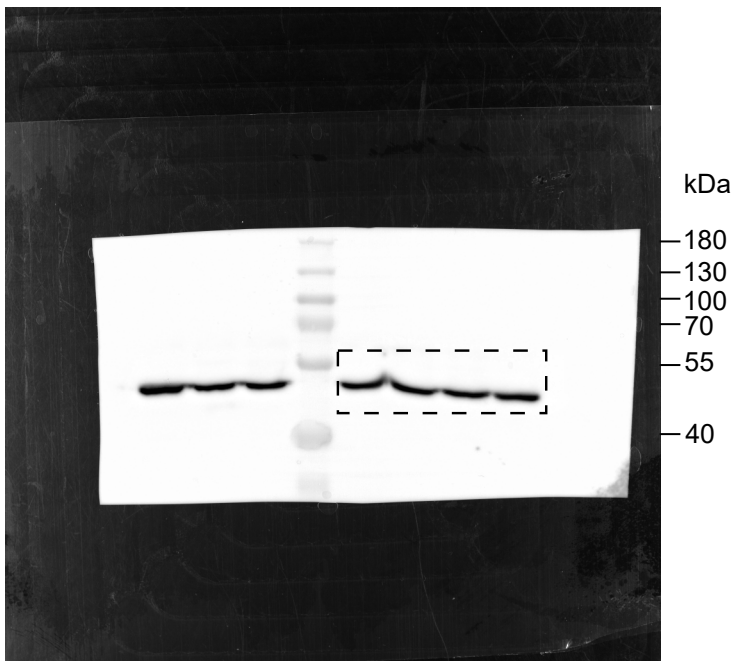

Supplement: Source Data Figure 4 [file EMS144463-supplement-Source_Data_Figure_4.pdf]

Figure 6b

Blot: mCherry

Blot: GAPDH

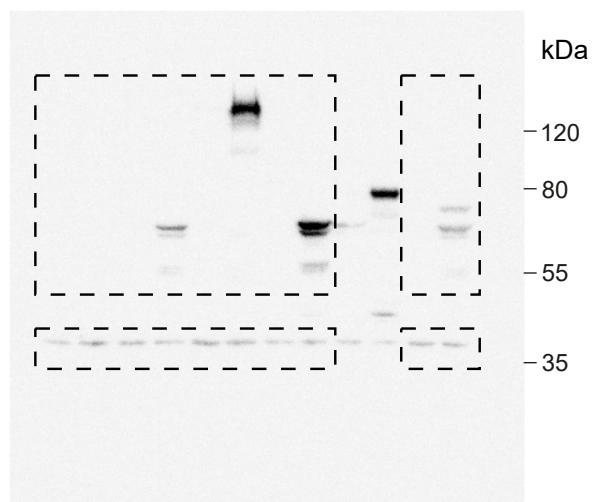

Supplement: Source Data Figure 6 [file EMS144463-supplement-Source_Data_Figure_6.pdf]
