# Supplementary material for: METALIC reveals interorganelle lipid flux in live cells by enzymatic mass tagging
Source: Nat Cell Biol. 2022 Jun 2;24(6):996–1004. doi: 10.1038/s41556-022-00917-9 (PMC9203272; doi:10.1038/s41556-022-00917-9)
Supplement: Supplementary file 1 [file 41556_2022_917_MOESM1_ESM.pdf]

nature portfolio

Corresponding author(s): Arun T. John Peter, Benoit Kornmann

Last updated by author(s): Apr 1, 2022

Reporting Summary

Nature Portfolio wishes to improve the reproducibility of the work that we publish. This form provides structure for consistency and transparency in reporting. For further information on Nature Portfolio policies, see our Editorial Policies and the Editorial Policy Checklist.

Statistics

For all statistical analyses, confirm that the following items are present in the figure legend, table legend, main text, or Methods section.

n/a	Confirmed	
☐	☒	The exact sample size (*n*) for each experimental group/condition, given as a discrete number and unit of measurement
☐	☒	A statement on whether measurements were taken from distinct samples or whether the same sample was measured repeatedly
☒	☐	The statistical test(s) used AND whether they are one- or two-sided<br>*Only common tests should be described solely by name; describe more complex techniques in the Methods section.*
☒	☐	A description of all covariates tested
☒	☐	A description of any assumptions or corrections, such as tests of normality and adjustment for multiple comparisons
☐	☒	A full description of the statistical parameters including central tendency (e.g. means) or other basic estimates (e.g. regression coefficient) AND variation (e.g. standard deviation) or associated estimates of uncertainty (e.g. confidence intervals)
☐	☒	For null hypothesis testing, the test statistic (e.g. *F*, *t*, *r*) with confidence intervals, effect sizes, degrees of freedom and *P* value noted<br>*Give P values as exact values whenever suitable.*
☒	☐	For Bayesian analysis, information on the choice of priors and Markov chain Monte Carlo settings
☒	☐	For hierarchical and complex designs, identification of the appropriate level for tests and full reporting of outcomes
☒	☐	Estimates of effect sizes (e.g. Cohen's *d*, Pearson's *r*), indicating how they were calculated

Our web collection on statistics for biologists contains articles on many of the points above.

Software and code

Policy information about availability of computer code

Data collection	Growth curves were obtained using the Clariostar software (version 5.4). Western blots were imaged using FusionCapt Advance FX7 software (version 17.03). Microscopy images were obtained using the Deltavision softWoRx software (version 6.5.2). Mass spectrometry data were obtained using the Thermo Q Exactive XCalibur software (version 4.0.27.10).
Data analysis	Image processing for microscopy and western blots: FIJI (Image J 1.53c)<br>Statisitics and Visualization: GraphPad Prism 8, Excel for Windows (Version 2108), RStudio (1.4.1103)<br>Lipidomics data analysis: Skyline software (version 21.2.0.369)

For manuscripts utilizing custom algorithms or software that are central to the research but not yet described in published literature, software must be made available to editors and reviewers. We strongly encourage code deposition in a community repository (e.g. GitHub). See the Nature Portfolio guidelines for submitting code & software for further information.

Data

Policy information about availability of data

All manuscripts must include a data availability statement. This statement should provide the following information, where applicable:

- Accession codes, unique identifiers, or web links for publicly available datasets
- A description of any restrictions on data availability
- For clinical datasets or third party data, please ensure that the statement adheres to our policy

Mass spectrometry data have been deposited to the MetaboLights metabolomics repository (dataset identifier MTBLS3415). Numerical source data (with all independent repeats) and unprocessed images of gels and blots are provided in the Source Data files. All other data supporting the findings of this study are available from the corresponding author on reasonable request. Source data are provided with this paper.

Field-specific reporting

Please select the one below that is the best fit for your research. If you are not sure, read the appropriate sections before making your selection.

☒ Life sciences ☐ Behavioural & social sciences ☐ Ecological, evolutionary & environmental sciences

For a reference copy of the document with all sections, see nature.com/documents/nr-reporting-summary-flat.pdf

Life sciences study design

All studies must disclose on these points even when the disclosure is negative.

Sample size	Experiments were performed a minimum of three times as a standard in the field.
Data exclusions	No data was excluded in this study
Replication	All data result from three independent experiments or experiments performed on three independent clones, unless otherwise stated with similar results. The number of replicates for each experiment is stated in the figure legends.
Randomization	For mass spectrometry runs, samples were randomized before queueing them in the autosampler. No other randomization was done, as the samples were derived from genetically identical, single colonies, and processed at the same time.
Blinding	No blinding was performed. All quantifications were performed without human interference.

Reporting for specific materials, systems and methods

We require information from authors about some types of materials, experimental systems and methods used in many studies. Here, indicate whether each material, system or method listed is relevant to your study. If you are not sure if a list item applies to your research, read the appropriate section before selecting a response.

Materials & experimental systems

n/a	Involved in the study	
☐	☒	Antibodies
☐	☒	Eukaryotic cell lines
☒	☐	Palaeontology and archaeology
☒	☐	Animals and other organisms
☒	☐	Human research participants
☒	☐	Clinical data
☒	☐	Dual use research of concern

Methods

n/a	Involved in the study	
☒	☐	ChIP-seq
☒	☐	Flow cytometry
☒	☐	MRI-based neuroimaging

Antibodies

Antibodies used	*mouse anti-Pgk1 antibody (Invitrogen, 459250, 1:3000 dilution), rat anti-RFP antibody (Chromotek, 5F8, 1:1000 dilution), mouse anti-FLAG antibody (Sigma, F1804, 1:1000), rabbit anti-mCherry antibdy, ab167453, 1:1000) and horseradish peroxidase-coupled secondary antibody (Bio-Rad, 170-6516; 1:10,000 dilution)*
Validation	*Antibodies were purchased from Invitrogen, Chromotek, Sigma, Abcam and have been validated in previous publications or by the manufacturer. mCherry antibody was validated using cell extract from HEK293 cells transfected with pFin-EF1-mCherry vector. For Pgk1, see https://doi.org/10.1038/s41556-021-00760-4 ; for RFP antibody, see https://doi.org/10.1089/hyb.2007.0555 and for FLAG antibody, see https://doi.org/10.1093/nar/gkab511*

Eukaryotic cell lines

Policy information about cell lines

Cell line source(s)	*HeLa cells were obtained from ATCC.*
Authentication	*HeLa cell line used in this study was not authenticated*
Mycoplasma contamination	The cell lines were routinely tested for mycoplasma contamination and the results were negative.
Commonly misidentified lines (See ICLAC register)	*HeLa cell line used in this study is not listed in the ICLAC database*
